# The Role and Potential Application of Antimicrobial Peptides in Autoimmune Diseases

**DOI:** 10.3389/fimmu.2020.00859

**Published:** 2020-05-08

**Authors:** Chunye Zhang, Ming Yang

**Affiliations:** ^1^Department of Veterinary Pathobiology, University of Missouri, Columbia, MO, United States; ^2^Department of Surgery, University of Missouri, Columbia, MO, United States

**Keywords:** autoimmune disease, antimicrobial peptides, immunomodualtion, therapy, diagnostic marker

## Introduction

Most of the antimicrobial peptides (AMPs) are small, cationic, and amphipathic peptides with <50 amino acids (1), which are also known as host defense peptides (HDPs). As listed in the Antimicrobial Peptide Database (APD) (2), over 2300 AMPs have been identified from animals. Above 130 AMPs have been tested in humans to date. They are constitutively and inducibly expressed in a variety of tissues where pathogens easily access, including gastrointestinal, urinary and respiratory tracts, and epithelial surfaces (3). Cathelicidins and defensins are two groups of AMPs broadly studied in humans. Defensins can be further subclassified into α-defensins and β-defensins by their secondary structures. AMPs have pleiotropic functions. The most well-known function is the antimicrobial activity against invading microorganisms, including bacteria, fungi, and enveloped viruses (4–6). Moreover, AMPs display other biological functions, such as lipopolysaccharide (LPS)-neutralization, wound healing, chemotactic activity, and immune modulation (3, 7–9). All their physiochemical characteristics, such as the molecular size, net charge, secondary or three-dimensional structure, and hydrophobicity, together determine their biological functions (7, 10, 11).

**Figure 1:**
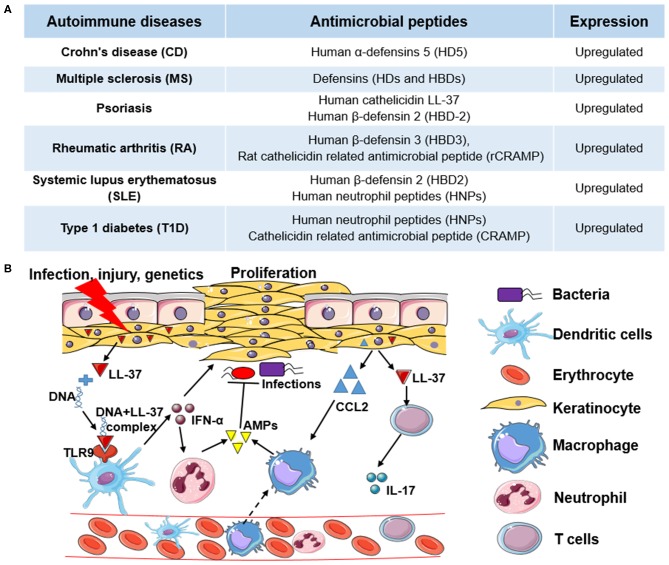
The role of antimicrobial peptides in autoimmune diseases and the molecular mechanism of human cathelicidin LL-37 in psoriasis. **(A)** Examples of antimicrobial peptides (AMPs) upregulated in different autoimmune diseases (ADs). **(B)** The molecular mechanism of human cathelicidin LL-37 in psoriasis. Under the pressure of infection, injury, or genetics, keratinocytes are activated to express high levels of cathelicidin LL-37, which binds with non-stimulatory self-DNA to form the self-DNA-LL-37 complexes to activate plasmacytoid dendritic cells (pDCs) to produce interferons (e.g., IFN-α) in a TLR9-dependent manner. IFN-α can not only stimulate the proliferation of keratinocytes but can stimulate the activation of neutrophils to express more AMPs. In addition, LL-37, as an autoantigen, can activate T cells to express cytokines (e.g., IL-17), which also stimulate the proliferation of keratinocytes. Chemokines (e.g., CCL2) expressed in keratinocytes, and other immune cells further induce monocyte or macrophage migration to promote inflammatory responses. In this process, LL-37 and other AMPs also have antimicrobial activity.

An occurrence of autoimmune diseases (ADs) happens when the host immune system attacks self-tissues with complex pathogenesis. The etiologies causing ADs are not fully understood. The genome-wide association studies (GWAS) reveal that genetic risk factors are involved in different ADs such as rheumatoid arthritis (RA), multiple sclerosis (MS), systemic lupus erythematosus (SLE), Type 1 diabetes (T1D), and celiac disease (12). The development of ADs in genetically-susceptible individuals is often triggered by environmental factors, including infectious agents, chemicals, and diets (13). The accumulating evidence shows that AMPs also play pivotal roles in autoimmune disorders. Most of the AMPs are induced or upregulated in the development of ADs, indicating their potential side effects (Figure 1A). For example, human cathelicidin LL-37 is positively associated with the pathogenesis of psoriasis, RA, and SLE (14, 15).

Microorganisms inhabit different areas, such as the intestine, skin, and oral sites. Bacteria, as the main part of microbes, have been considered to play a vital role in the pathogenesis of ADs at those organs (16). For instance, Manfredo Vieira et al. found that translocation of a gut bacterium *Enterococcus gallinarum* to the liver or other organs can trigger autoimmune responses in mice with a genetic background of predisposing to lupus-like autoimmunity (17). Recent metagenomics studies also reveal that dysbiosis of gut microbiota impacts the progression of ADs in infants (18, 19). In addition, dysbiosis of gut microbiota induced by environmental factors is able to change the expression of AMPs, which indicates the crosslink between gut microbiota and ADs. Herein, in this review, we discuss the potential effects of AMPs in different ADs and their potential roles as diagnostic and therapeutic agents.

## The Potential Utility of Antimicrobial Peptides as Biomarkers for Autoimmune Diseases

AMPs have displayed multiple roles in the innate and adaptive immunity. Accumulating evidence suggests that AMPs could be diagnostic markers for ADs. For instance, the expression of human neutrophil peptides (HNPs) 1–3 significantly increased in the synovial cavity of patients with RA (20). The significant correlation between the joint erosion and the presence of HNPs suggests they might be used as diagnostic markers for RA. In addition, the plasma level of HNP 1-3 was significantly increased in T1D patients compared to healthy controls (21). Serum levels of HBD-2 and HNPs are also elevated in SLE patients (22). Cathelicidin LL-37 and autoantibodies to LL-37 were elevated in patients with psoriatic arthritis (PA), which was correlated with clinical inflammatory markers (23). In the following context, we will describe the role of AMPs in different ADs and how AMPs function in their development.

## Psoriasis

Psoriasis is a chronic autoimmune skin disease resulting from genetic, epigenetic, environmental, and lifestyle factors (24), which affects 125 million people worldwide, according to the World Psoriasis Day consortium (https://ifpa-pso.com/our-actions/world-psoriasis-day). Aberrant production of interferons (IFNs) in plasmacytoid dendritic cells (pDCs) is a major pathogenic effector in psoriasis, which triggers the activation of T cells. As shown in Figure 1B, antimicrobial peptide LL-37 secreted by keratinocytes of lesional skin binds with non-stimulatory self-DNA to form the self-DNA-antimicrobial peptide complexes. These complexes can translocate into pDCs to activate IFN-α production in a TLR9-dependent manner (25), which promotes the progression of psoriasis. LL-37 also can bind with self-RNA from dying cells to form self-RNA-LL-37 complexes, which can trigger the secretion of IFN-α in pDCs by activating TLR7 and TLR8 to exacerbate psoriasis (26). Lande et al. also reported that LL-37 could directly trigger T cell activation as an autoantigen in psoriasis (27). In addition to keratinocytes, increased infiltrating neutrophils are another group of cells that primarily produce AMPs in psoriasis. Meanwhile, neutrophils can induce human β-defensin 2 (HBD-2) expression in keratinocytes of psoriatic skin by forming neutrophil extracellular traps (NETs) (28). Except for neutrophils and pDCs, other AMP-producing innate lymphocytes such as natural killer T (NKT) cells and natural killer (NK) cells also play critical roles in the pathogenesis of psoriasis (29). Overall, AMPs with AMP-producing cells play an essential role in the pathogenesis of psoriasis through both the innate and adaptive immune responses.

## Rheumatic Arthritis

Inflammatory arthritis (IA) is an autoimmune disease characterized by synovial hyperplasia, which can be driven by genetic and environmental factors. The proteomics and peptidomics analysis of synovial fluid (SF) showed that AMPs played an essential role in the molecular underpinnings of IA (30). For example, the expression of human α-defensin 3 (HD-3) is significantly upregulated in the SF of IA patients. RA is the most common and destructive IA, a well-known chronic autoimmune disorder (31). Neutrophils contribute to the pathogenesis of RA by secreting pro-inflammatory cytokines and chemokines, forming NET, and generating autoantigens to drive tissue damage (32). The rat cathelicidin related antimicrobial peptide (rCRAMP) was significantly upregulated in the joints of rats with pristane-induced arthritis (PIA) (33). The expression of rCRAMP was primarily from neutrophils and macrophages (14). In the SF of human RA patients, CD163^+^ macrophages and CD66^+^ granulocytes are the primary cells expressing LL-37, which has been discussed to display a boosting effect in psoriasis. On the other side, treatment with anti-rheumatic agents, such as adalimumab and etanercept, caused a significant reduction of LL-37 expression, which indicates that there is an association between the expression of LL-37 and RA disease severity (34).

## Type 1 Diabetes

Type 1 diabetes (T1D) is an autoimmune disease resulting from the destruction of insulin-producing pancreatic β-cells (35). The upregulation of cathelicidin related antimicrobial peptide (CRAMP) in the intestine of diabetes-prone BioBreeding (BBdp) rats was observed during the development of diabetes. The gut microbiota and dietary antigens impact the incidence of T1D both in animals and humans (36, 37). Gut microbiota can control the expression of CRAMP in pancreatic β-cells through the production of short-chain fatty acids (38). These studies indicate that modulating gut microbiota can be applied to reduce the development of autoimmune diabetes through the change of AMP expression.

## The Role of Gut Microbiota in Other Autoimmune Diseases

In addition to T1D, dysbiosis of gut microbiota may also trigger other autoimmune diseases, such as MS and SLE (39, 40). Emerging evidence shows that probiotic treatment may improve the prognosis of autoimmune diseases by modulating gastrointestinal symptoms and multi-organ inflammation (41). One mechanism of efficacy of probiotic therapy is to modulate the secretion of AMPs in the intestine (42, 43). Several other approaches, including AMPs, antibiotics, fecal microbiota transplantation (FMT), and prebiotics, have been applied to regulate the gut microbiota in clinical or preclinical studies to treat autoimmune disorders (44). Overall, dysbiosis of gut microbiota may trigger ADs in genetically susceptible persons, and modulating gut microbiota by antimicrobial intervention can be applied to prevent the development of ADs.

## The Beneficial Role of Antimicrobial Peptides in Autoimmune Diseases

AMPs bridge the connection of innate and adaptive immune responses with a broad spectrum of microbicidal activity. Hence, they are double-edged swords in autoimmune disorders. In addition to the above-mentioned side effect, exogenous AMPs treatment can reduce the symptoms of AD. For instance, administration of scolopendrasin IX, an AMP that was identified from centipede *Scolopendra*, could inhibit the expression of inflammatory cytokines through formyl peptide receptor 2 (FPR2), such as tumor necrosis factor alpha (TNF-α) and interleukin 6 (IL-6). Administration of scolopendrasin IX also can reduce the clinical phenotype of RA in a mouse model induced by transgenic K/BxN mice serum (45). Treatment with rhesus θ-defensin 1 (RTD-1), a family of macrocyclic peptides exclusively expressed in monkey, suppressed the joint disease progression and restored limb mobility in a rat model of rheumatoid arthritis (46). Furthermore, the *in vivo* treatment with the LL-37 peptide in BBdp rats promoted β-cell neogenesis and upregulation of potentially beneficial gut microbes, resulting in a reduction of symptoms of autoimmune diabetes (36). Injection of mouse β-defensin-14 (mBD14) ameliorated the central nervous system inflammation and pro-inflammatory cytokines and cytotoxic T cells in the model of experimental autoimmune encephalomyelitis. *In vitro* study further showed that human β-defensin-3, the human ortholog of mBD14, shifted non-regulatory CD4^+^CD25^−^ T cells into a regulatory phenotype with the expression of Treg markers, such as Foxp3 and GARP (47). Overall, these research results indicate that there are some beneficial effects of AMPs in AD treatment.

## Summary

Pro-inflammatory cytokines and chemokines play a pivotal role in the pathogenesis of autoimmune diseases (48). The exposure of pro-inflammatory cytokines, such as TNF-α and LPS, could significantly increase the expression of HBD-2 in cultured chondrocytes (49). Furthermore, AMPs secreted from activated chondrocytes, keratinocytes, or neutrophils can further impose the inflammatory response by chemoattracting more monocytes or other immune cells, advancing tissue damage manifested in patients with ADs (50). The loop of initial proinflammatory response-AMP expression-immune cell infiltration plays a crucial role in the progression of autoimmune disorders. Recently, gut microbiota has been shown to play a key role in the pathogenesis of ADs. For instance, lower *Firmicutes*/*Bacteroidetes* ratio has been shown in SLE (39). Meanwhile, changes in the gut and oral bacteria are implicated in the pathogenesis of other ADs, such as RA, systemic sclerosis, and anti-phospholipid syndrome. Modulations of gut microbiota through probiotics or fecal transplantation (FMT) are considered a promising therapeutic strategy for ADs (39). AMPs, as the product and modulator of gut microbiota, can also be applied to defense against the infection and balance the dysbiosis of gut microbiota, which is beneficial to the treatment of ADs. Currently, the roles of AMPs in the pathogenesis of autoimmune diseases are better understood. The clinical trial results for investigation of AMPs in autoimmune disorders are expected. A combination of AMPs with other non-aggressive biomarkers (e.g., TNF-α) could improve the diagnosis of autoimmune disorders in the early stage. Additionally, AMPs are promising targets to design personalized treatment for patients with ADs.

## Author Contributions

CZ and MY conceived the opinion and wrote the manuscript.

## Conflict of Interest

The authors declare that the research was conducted in the absence of any commercial or financial relationships that could be construed as a potential conflict of interest.
